# New methods for oscillation analyses push new theories of discrete cognition

**DOI:** 10.1111/psyp.13827

**Published:** 2021-05-04

**Authors:** Mikael Lundqvist, Andreas Wutz

**Affiliations:** ^1^ Department of Clinical Neuroscience Karolinska Institute Stockholm Sweden; ^2^ Picower Institute for Learning & Memory Massachusetts Institute of Technology Cambridge MA USA; ^3^ Centre for Cognitive Neuroscience University of Salzburg Salzburg Austria

## Abstract

Classical ways of analyzing neural time series data has led to static views on cognition, in which the cognitive processes are linked to sustained neural activity and interpreted as stationary states. The core analytical focus was on slow power modulations of neural oscillations averaged across many experimental trials. Whereas this custom analytical approach reduces the complexity and increases the signal‐to‐noise ratio, it may disregard or even remove important aspects of the underlying neural dynamics. Novel analysis methods investigate the instantaneous frequency and phase of neural oscillations and relate them to the precisely controlled timing of brief successive sensory stimuli. This enables to capture how cognitive processes unfold in discrete windows within and across oscillatory cycles. Moreover, several recent studies analyze the oscillatory power modulations on single experimental trials. They suggest that the power modulations are packed into discrete bursts of activity, which occur at different rates and times, and with different durations from trial‐to‐trial. Here, we review the current work that made use of these methodological advances for neural oscillations. These novel analysis perspectives emphasize that cognitive processes occur in discrete time windows, instead of sustained, stationary states. Evidence for discretization was observed for the entire range of cognitive functions from perception and attention to working memory, goal‐directed thought and motor actions, as well as throughout the entire cortical hierarchy and in subcortical regions. These empirical observations create demand for new psychological theories and computational models of cognition in the brain, which integrate its discrete temporal dynamics.

## Abstract 2

Cognition is an ongoing and dynamic process. Yet, classic theories emphasize a stagnant view on the mind. We review recent methodological advances for oscillation analyses, which capture dynamic aspects of cognition and emphasize its discrete temporal dynamics.

## 1 INTRODUCTION

In the last decades cognitive neuroscience research has fostered a stagnant view on the mind. Historically, we have used a reductionist approach and studied cognition by means of static stimuli and single isolated responses, identified its functional properties in stationary mechanisms and located its neural correlates in spatially defined brain maps. Experiences and behaviors in the real world, however, are ongoing and unfold dynamically *over time*. Indeed, we often encounter dynamic situations in everyday life. A simple example is the task of crossing a busy street. Perceiving and predicting the upcoming vehicles requires information integration over hundreds of milliseconds or even seconds, often including the combination of motion signals across occlusion or changes in retinal position caused by eye movements. Another paramount example is team sports, like basketball or football, in which the spatiotemporal coordinates of the teammates, the opponents and the ball must be updated from moment‐to‐moment and at the same time complex game rules (e.g., “offside‐rule”) must be understood and followed upon.

In order to sense, analyze, and understand such dynamic situations, we need a dynamic mind with flexibly interacting perception, attention, and working memory systems. When viewed from a real‐time perspective: Perception goes beyond purely spatial definitions in retinotopic coordinates (DeYoe et al., 1996). Attention requires more than selecting prioritized spatial locations or objects (Posner, 1980). And, working memory cannot be reduced to spatial resources or space‐based saliency maps (Alvarez & Cavanagh, 2004; Todd & Marois, 2004). Instead in real time: perception transforms continuously changing signals into spatiotemporally coherent objects, scenes, and events (for reviews see Ogmen & Herzog, 2010; Wutz & Melcher, 2014). It achieves this impressive feat despite of often very abrupt and transient input changes due to object‐ or self‐motion (Otto et al., 2010). In fact, the retinal image is re‐established and updated about 3–4 times per second due to gaze shifts via saccadic eye movements (Otero‐Millan et al., 2008). Likewise from a real‐time perspective: attention dynamically and periodically shifts its processing focus at a similar rate to overt exploration via ocular‐motor commands (Landau & Fries, 2012; for review see VanRullen, 2013). And working memory, the brain's *online* workspace, may not just store content in a sustained, static format. Instead, working memories need to be flexibly controlled and manipulated, in order to understand complex relationships like agency and causation, allow for abstraction and generalization beyond physical appearance and prospectively support intelligent, goal‐directed behavior (Lundqvist et al., 2016; Wutz et al., 2018; for reviews see Miller et al., 2018; Nobre & Stokes, 2019; Zeithamova et al., 2019).

In light of the highly dynamic nature of cognition, a key challenge for cognitive neuroscience is to come up with new theories that emphasize its dynamic aspects and are derived from new, dynamic analytic approaches for neural signals. In experimental psychology and cognitive science, empirical and theoretical work on the temporal aspects of perception, attention, and working memory have a long history (e.g., Di Lollo, 1980; Mach, 1865; Philips & Baddeley, 1971; Sperling, 1960; Stroud, 1956; Wundt, 1899). However, systematic investigations on the temporal organization of cognition *in the brain* are just picking up pace (for reviews see Fiebelkorn & Kastner, 2019; Öğmen & Breitmeyer, 2006; VanRullen, 2016). Electrophysiological measures of neural activity (e.g., electro‐encephalography (EEG), magneto‐encephalography (MEG), electro‐corticography (ECoG), and intracranial single‐cell or multi‐electrode recordings) are predestined to push dynamic theories of cognition, because they provide the necessary temporal resolution on the millisecond scale to track neural signals in real time.

Current perspectives highlight that neural processing relies on iterative and recurrent routing of information flow across distributed brain networks (for reviews see Buzsaki, 2006; Varela et al., 2001). Mutual excitation and inhibition via synaptic interactions between groups of neurons, so called neural ensembles, promotes synchronized firing rates across neurons and gives rise to oscillations in their mean local field potential (for review see Wang, 2010). In this view, oscillations are a hallmark signature of brain networks and their information processing. Moreover, oscillations are a prime candidate to capture the dynamic nature of cognitive processes in the brain, because they are ubiquitous in neural recordings and they provide time windows for communication to the sensory environment and between brain areas (for review see Fries, 2015). Oscillations often occur within specific canonical frequency bands in the brain (Table 1). In recent years, connecting these frequency‐specific neural oscillations to cognitive functions has become a strong trend in cognitive neuroscience (see *Google Scholar* search results in Table 1). The plethora of links found in previous research suggests a strong relationship between oscillations and functions. But it also demonstrates that—up to now—there is no consistent one‐to‐one mapping of functions onto oscillations. Virtually all frequency bands of interest are associated with several cognitive functions, and vice versa.

**TABLE 1:** Oscillations in the brain and their links to cognition (quantified in * Google Scholar search results on 27/10/2020) function

		Frequency (Hz)	Cycle time (ms)	Perception	Attention	WM
Oscillation	Theta	3–7	140–300	35,500*	75,200	36,900
	Alpha	8–12	80–120	68,500	149,000	55,500
	Beta	15–35	30–60	62,000	112,000	55,100
	Gamma	>40	<25	60,100	171,000	53,100

One key factor is the typical analytical focus on sustained increases in oscillation amplitude/power for neural time series data. Whereas oscillations by themselves suggest nonstationary dynamics, their interpretation as sustained activity leads one to think of stationary brain states and cognitive processes. This mismatch has generated the urge to develop new analytical approaches for neural oscillations highlighting their nonstationary dynamics and their functional role for dynamic aspects of cognition. One promising methodological avenue is the investigation of temporal parameters of neural oscillatory signals, that is, its instantaneous frequency and phase timing. In particular, the combined investigation of the temporal profile of oscillations together with time‐sensible psychophysics, which precisely control the temporal relationships between successive sensory stimuli, offers great potential for dynamic views on perception and attention. A second recent methods innovation is the analysis of burst‐like dynamics of oscillatory activity. Burst event analyses reveal neural activity increases that occur at different rates, times and with different durations from trial‐to‐trial. Thus, burst events are more diagnostic of dynamic, nonstationary activity changes in neural signals, and better capture the flexible use of working memories and intelligent, goal‐directed thought, and motor action. It also allows for more precise investigations of timing between behavior and neurophysiology. Here, we review these recent methods advances in oscillation analyses, how they highlight the intrinsic nonstationary characteristics of oscillations and how they push for a paradigm shift from classic static to radically new theories of discrete cognition.

## 2 OSCILLATORY FREQUENCY AND PHASE ANALYSES SUGGEST “DISCRETE INTEGRATION WINDOWS”—THEORIES OF PERCEPTION AND ATTENTION

Discrete perception and attention in the brain are a longstanding idea that goes back to early investigations in experimental psychology on “perceptual moments” (e.g., Haber & Standing, 1970; Shallice, 1964; Stroud, 1956), but it was for a long time neglected in cognitive neuroscience (for review see VanRullen & Koch, 2003). By now, it has become a leading view again due to a recent boom in reports from electrophysiology that connects its typical time frames (≈100–200 ms) to brain oscillations (for review see VanRullen, 2016). Discrete cognition theories make much sense from a computational perspective, because discretization reduces complexity and sets the ground for cascade‐like information transfer in the brain. However, discrete processing is not readily compatible with our phenomenological experience of a stable and continuous sensory world. We do not perceive a scattered series of still images interleaved with blank epochs (except for rare neurological cases; i.e., Akinetopsia; Sacks, 2004). Moreover, discrete sampling in retinotopic visual coordinates would leave motion smear in the image plane, when the processed inputs change faster than the sampling refresh rate (the so‐called “moving ghost problem”; Ogmen & Herzog, 2010). However, we typically perceive clear and un‐blurred objects at well‐defined locations (Burr, 1980; Burr et al., 1986). How can discrete perception and attention systems achieve continuity and stability concurrently in the sensory world?

New analysis strategies that investigate the temporal relationships between the nonstationary dynamics of neural oscillations and sensory signals shed light on this fundamental question for real‐time cognition. They suggest that perception and attention are organized into discrete, multiplexed temporal windows in the brain, in order to simultaneously achieve input integration (supporting continuity) and individuation (supporting stability). Classic theories of discrete perception often describe it with surveillance or video camera metaphors, typically introducing some kind of input sampling at a certain periodic phase and input intermittency at the opposite phase. Sampling and intermittency can either deal with inputs from the external world or from internal representations, each coming with its respective issues with respect to motion blur or perceived continuity. On the neural level, however, discretization is typically thought to result from the intermittent and synchronous firing of excitatory and inhibitory neural ensembles, which gives rise to oscillations in their mean activity. Current interpretations of discrete processing in terms of “integration windows” focus on the integration processes between this ongoing discretization machinery due to transient neural network activity and input signals from downstream sensory neurons. They are framed around the idea that oscillatory cycles in the brain constitute discrete event boundaries for input integration (which is in line with earlier theories, Gho & Varela, 1988; Kristofferson, 1967; Varela et al., 1981). In short: Two signals falling within the same cycle are integrated into one event, whereas temporal segregation occurs when they fall into two different cycles. “Discrete integration windows”—theories are consistent with theoretical perspectives, in which perception per se relies on discrete temporal parsing of successive sensory inputs, rather than dealing with discrete contents sampled from external or internal representations. As noted already by Von Helmholtz (1867) such temporal windowing operations could serve as a possible solution for perceived continuity, when moments in perception show hysteresis, such that successively sampled images partly overlap and their properties are temporally integrated (i.e., a sliding integration window). Historically, sensory memory outlasting the actual stimulus duration and its temporal integration with successive stimuli are well‐described phenomena in visual psychophysics (e.g., Coltheart, 1980; Di Lollo, 1980; Loftus et al., 1992). Temporal integration in sensory memory is typically investigated in fusion‐, integration‐, or masking paradigms by experimentally manipulating the temporal relationships between (typically two) successive sensory stimuli (for classic examples see Crozier & Wolf, 1941; Scheerer, 1973a, 1973b; for reviews see Enns & Di Lollo, 2000 and Breitmeyer & Öğmen, 2006).

Consequently, recent neuroscience work has combined the analysis of the phase and frequency of neural oscillations with “old‐school” psychophysics methods about temporal integration. Typically, this work aims for precise control of the temporal relationships between successive sensory stimuli with behavioral performance at a given psychometric threshold (see also the special section “Advantages of phase and frequency analyses”). For example, periodically presented stimuli can entrain neural oscillations in the alpha band (~10 Hz) and modulate the detection rate of subsequent near‐threshold stimuli (Mathewson et al., 2010; see Lundqvist et al., 2013 for reproduction in computational networks). Moreover, the participants’ individual frequency of alpha oscillations has been shown to predict two‐flash fusion thresholds (Samaha & Postle, 2015) and multisensory integration in the sound‐induced double‐flash illusion (Cecere et al., 2015). In recent work, participants switched between task demands for temporal integration versus segregation of two successive visual displays while the stimuli and performance were held constant across trials. They showed that the visual system strategically modulates its oscillatory alpha frequency to adjust its temporal resolution according to task goals (Wutz et al., 2018). The key insights from this research line are that temporal integration in vision occurs for speeds of at least 10 images per second, that it is correlated with the frequency of alpha oscillations over occipital‐parietal regions, and that it is under top‐down control by the perceiver.

**FIGURE 1:**
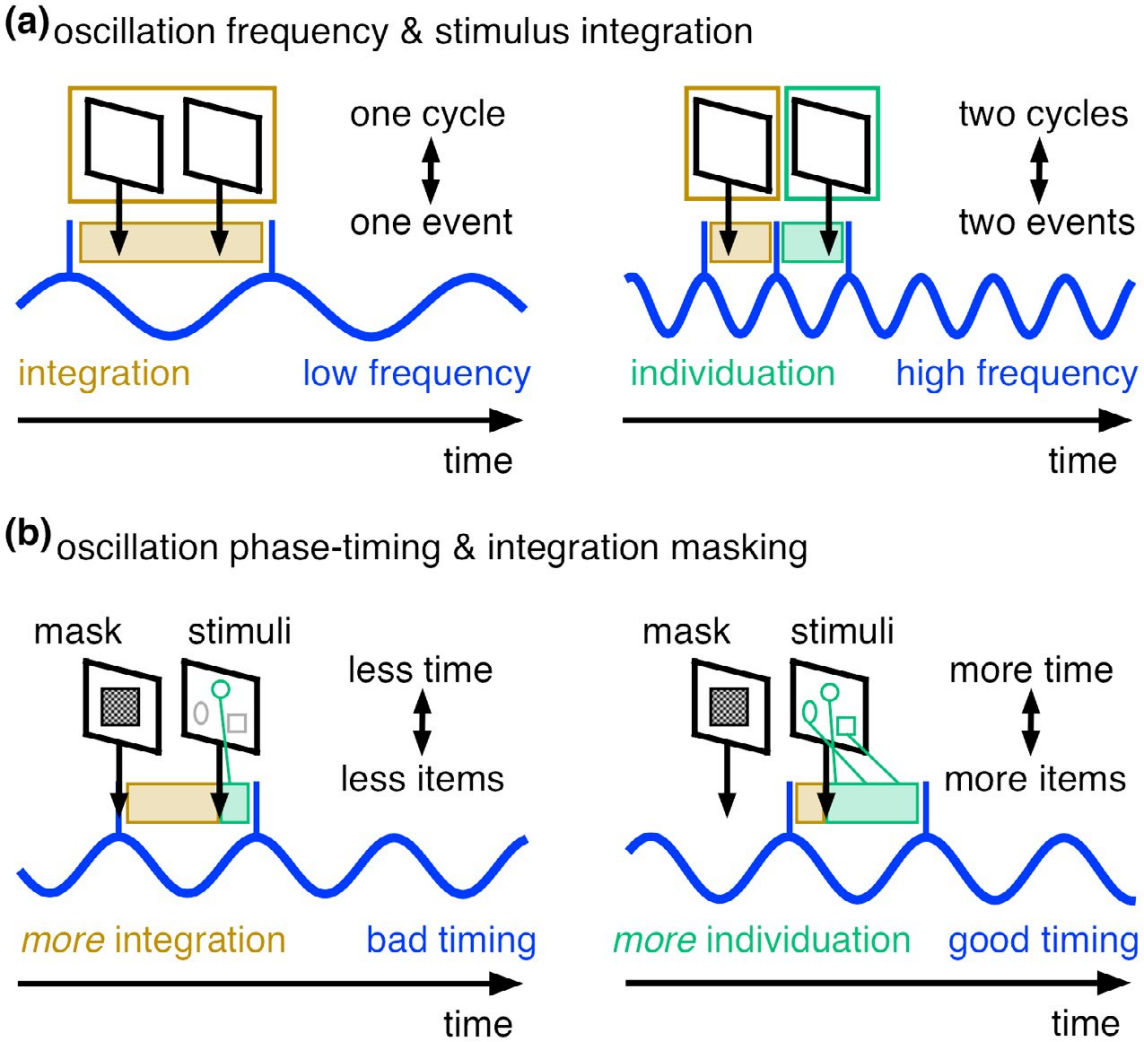
Oscillations and “discrete integration windows.” (a) Schematic depiction for the relationship between oscillatory frequency and stimulus integration. The onsets of two successive stimuli are integrated into one perceptual event when they fall into the same neural oscillatory cycle. In contrast, the two sensory stimuli are individuated into two perceptual events when they fall into two different neural cycles. Consequently, lower oscillatory frequencies lead to more integration and higher frequencies lead to more individuation by altering the probability that the two stimuli fall into the same versus different cycles (Wutz, Melcher, et al., 2018). (b) Schematic depiction for the relationship between oscillatory phase‐timing and integration (forward) masking. Integration masking occurs when sensory stimuli are processed within the same neural oscillatory cycle. Consequently, the neural phase timing with respect to mask‐ and stimulus onsets influences the perceptual outcome (Wutz et al., 2014). Individuation fails at bad neural timing because of greater mask‐stimulus integration and less time to individuate the stimulus into items within a cycle (one green item and two unprocessed gray items). In contrast, many items are correctly individuated (three green items) when there is less mask‐stimulus integration and more time to process the stimulus within a cycle. Note that in all cases the two stimuli are equally distant in physical time. Instead, their psychological interpretation (in terms of same or different events) and information content (i.e., the number of objects per event) depends on neural timing (i.e., the frequency and phase of neural oscillations)

From an analytical point of view, investigating time‐varying estimates of the frequency of oscillations, in addition to their power, is a major methods advance. The instantaneous frequency is defined as the time rate of change (i.e., its temporal derivate) of the instantaneous phase angle (Cohen, 2014; Samaha & Postle, 2015). By definition, one cannot specify the frequency of a signal (i.e., the rate over time) at one specific time point and the phase angle time series is prone to noise. Thus in order to obtain stable frequency estimates around a given time point, but at the expense of temporal smoothing, it is computed in several, differently sized median filter windows and then, median averaged over the filters (see Box 1). Conceptually, this recent work on instantaneous oscillatory frequencies suggests that faster frequencies relate to a higher temporal resolution, because of the increased probability that two stimuli fall into two different cycles (Figure 1a).

BOX 1 **Instantaneous frequency analysis pipeline**

1. Band‐pass filter raw time series in frequency band of interest for each trial
2. Hilbert‐transform band‐limited signals
3. Calculate differential of instantaneous Hilbert‐phase estimate
4. Apply Median‐Filter several times with differently sized windows
5. Compute Median of Medians over windows

Further evidence for discrete processing in terms of temporal “integration windows” embedded in neural cycles comes from masking paradigms, in which the visibility of one target stimulus is reduced by the presence of another mask stimulus. For example, for forward integration masking, masking effects are typically explained by trailing sensory traces from the mask temporally overlapping and integrating with the following target onset and its associated sensory trace (Di Lollo, 1980). Integration masking procedures effectively enable to slice sensory memory for the target into smaller time units by systematically varying short and long temporal jitter between the onsets of the mask‐ and the target stimulus displays. It is thus ideal to investigate the time course of early structuring computations in the visual image (i.e., visual routines (Ullman, 1984), object individuation (Xu & Chun, 2009)), whose outputs in the form of “object files” can provide visual stability over time (Kahneman et al., 1992). By definition, object individuation requires selecting features from a scene, binding them into an object and individuating it from other objects and the background (Xu & Chun, 2009). Classically, object individuation was assumed to happen instantaneously “at a glance” (Jevons, 1871; Kaufman et al., 1949) and its capacity was mainly attributed to spatial factors (Alvarez & Cavanagh, 2004; Todd & Marois, 2004). However, using integration masking together with MEG recordings revealed that individuation capacity increases in steps with more time left in sensory memory and that it depends on neural phase timing (Wutz et al., 2012; Wutz & Melcher, 2013; Melcher et al., 2020). This suggests that individuation capacity is reached with a certain bandwidth over time. The rate of data transfer defaults to approximately one object per 25 ms and it increases up to capacity limits (≈ 4 objects) within 100 ms (for review see Wutz & Melcher, 2014). Moreover, correct individuation was reflected in precisely timed phase resetting in the alpha frequency band (~10 Hz) during mask‐ and target‐stimulus processing over occipital‐parietal cortex (Wutz et al., 2014; see Figure 1b for a schematic depiction and the special section “Advantages of phase and frequency analyses”). Individuation was only linked to neural phase timing for short temporal jitters, when the onsets of the mask and target stimuli could fall into one alpha cycle (<100 ms). Instead for longer temporal jitters between mask‐ and target displays (>100 ms), individuation depended on more sluggish alpha/beta power modulations prior to stimulus onset. Moreover, the evoked response to target stimuli was systematically shifted by a constant delay of ~100 ms relative to the mask‐evoked response when there was a short temporal jitter between the mask‐ and target displays (<100 ms). This shift was absent for longer temporal jitters between mask‐ and target displays (>100 ms), potentially indicating that only for short temporal jitters target stimulus processing was referred to the next processing cycle.

In sum, this body of work suggests that temporal integration in discrete neural cycles influences building and structuring operations in intermediate‐level vision (i.e., object individuation), rather than judgments about stimulus detection, its temporal duration or simultaneity. This is an important point, since psychophysical findings about the temporal aspects of perceptual content (i.e., whether a stimulus is seen or missed, for how long it is seen, or whether two stimuli are seen at the same time or sequentially) are logically independent from the temporal building blocks of perception *itself* (Herzog et al., 2016, 2020; van Wassenhove, 2017). As known from experimental psychology, the selective transfer of volatile features into reportable objects depends on the buffering of inputs into sensory memory (Gegenfurtner & Sperling, 1993; Sperling, 1960). Thus, sensory integration windows in neural cycles may support the construction of a stable object‐based reference frame, in which successive inputs can be mapped onto stable, invariant, and explicit object representations, such that their recent spatial‐temporal history can be reviewed (Kahneman et al., 1992). Specifically, an object‐based reference frame in nonretinotopic coordinates is a prerequisite for solving the “moving ghost problem” for objects in motion and for perceptual stability (Ogmen & Herzog, 2010; Otto et al., 2010). Consistent with this perspective, recent empirical evidence reports object formation as a flexible and dynamic process depending on temporal variables, such as motion velocity (Alvarez & Franconeri, 2007) and sampling speed (Holcombe & Chen, 2013). Current theoretical models suggest that objects are translated into a temporal code according to the cycle‐phase of neural oscillations (Jensen et al., 2014; Lisman & Jensen, 2013; Wutz & Melcher, 2014).In principle, this solution offers a possible means to translate continuous functions (like oscillations) into discrete representations (like objects). Such models are also supported by single‐cell physiological evidence (Axmacher et al., 2010; Siegel et al., 2009). Their common computational principle is that features of individual objects and spatial locations are processed via feed‐forward activation, possibly reflected in spiking activity or gamma oscillations in primary sensory areas. However, the entire perceptual event, involving the construction of discrete objects in a stable scene layout, is computed over a longer time window. Each processing window can serve as a buffer to regulate input integration and individuation. Using such temporal windows, cognition might thus accomplish temporal continuity and stable object read‐out virtually in real time by means of temporal multiplexing (Panzeri et al., 2010).

The current state‐of‐the‐art suggests that discrete perception and attention systems compute “content in windows” implemented within neural cycles. Important open questions are how those windows are aligned to events in the external world, how different windows are synced to each other, and how discrete cognition operates across successive windows/cycles. One possibility might be that the brain's timing is synced to processing transitions via phase resetting (e.g., when new inputs arrive at stimulus onset or at gaze shifts). Brain processes in different areas can be timed via consistent phase relationships (e.g., Fries, 2015; Klimesch et al., 2007; Rassi et al., 2019) and evidence for a functional role of phase resetting has been reported in EEG/MEG activity after stimulus onset over occipital‐parietal cortex (Makeig et al., 2002; Wutz et al., 2014) and in time‐resolved behavioral measures after focus shifts (Landau & Fries, 2012; Wutz et al., 2016). Thus, phase‐timed coordination might reflect an optimal strategy to synchronize perception and attention systems with neural processing. Moreover, it becomes increasingly clear that multiple neural oscillations on different time scales underlie the spatial‐temporal organization of perception and attention. As highlighted above, alpha oscillations (~10 Hz) are thought to support integration and individuation in sampling windows over faster time scales (≈100ms) and at the same spatial location (e.g., in fusion paradigms). Theta oscillations (~5 Hz), moreover, operate sequentially over longer time scales (≈200 ms) and across space (e.g., for apparent motion; Ronconi et al., 2017). Recent evidence also suggests that the switching between multiple objects is supported by the temporal coordination of alpha and theta oscillations in the brain (Jia et al., 2017). Thus, alpha and theta oscillations might be coupled to resolve exploitation–exploration conflicts arising from real‐time processing. This view is also consistent with emerging theoretical perspectives, which describe cognition to alternate between two different states for sensory (sampling) and motor (shifting) processes at opposite rhythmic phases in the brain (Fiebelkorn & Kastner, 2019). Moreover, gamma oscillations (> 40 Hz) are a neural correlate of feed‐forward encoding and selectively attending to specific sensory features (Fries et al., 2001). The fast feed‐forward activation of specialized motion‐selective units might be a primary reason for our ability to detect change and motion on the millisecond scale despite discrete processing over longer windows. In sum, this multitude of relevant neural oscillatory frequencies supports theoretical models, which suggest that different aspects of our sensory world are computed in parallel and on different time scales (Dennet & Kinsbourne, 1992; Herzog et al., 2016). Given that different neural oscillations such as theta, alpha, and gamma appear to be generated by distinct neural populations (Bastos et al., 2018; Bollimunta et al., 2011; Buffalo et al., 2011), diversification of their functions may arise naturally within the cortical network. Of course, these “multiple‐drafts” of perceptual reality need to be integrated and combined at some point. A typical example for such integration processes is post‐diction, in which the presence of a later event alters the perceptual representation of a former event. Post‐dictive integration effects often occur over time scales, which extend beyond the typical time range of alpha cycles (for review see Herzog et al., 2020). In order to better understand such complex phenomena, one key challenge for future research will be to apply the described analytical tools for neural oscillatory phase and frequency across multiple different oscillations and carve out how different processing streams are integrated and multiplexed with respect to each. Moreover, it will be crucial to determine how instantaneous frequency and phase analysis can help to understand how processing evolves across successive cycles over time. One promising approach are burst extraction methods (see the special section: “Advantages of burst analyses”). Bursts typically extend over multiple successive cycles and thus may reflect a higher‐order organizing principle of discrete cognition in the brain beyond one cycle of a particular frequency.

## 3 SPECIAL SECTION: “ADVANTAGES OF PHASE AND FREQUENCY ANALYSES”

Extracting the instantaneous frequency and phase from time series data provides several advantages.

1. It allows investigating the fine‐grained, nonstationary temporal structure of oscillations. Classically, neural time series data analyses focuses on oscillatory power, which often shows slower modulations over several hundreds of milliseconds or even seconds. Instead, instantaneous frequency and phase analyses reveal the intrinsic nonstationary dynamics of oscillations (i.e., their rate of change between peaks and troughs).
2. Oscillations signal network interactions in the brain. Thus, the instantaneous frequency and phase informs about the speed and the timing of this neural network communication. This can help to develop more accurate representations how information processing evolves across the cortex and over time.
3. An important asset is the possibility to pair instantaneous frequency and phase measures with precisely timed presentations of successive sensory stimuli (typically two). This allows answering whether perceptual processes depend on the timing of the stimulus onsets relative to oscillatory cycles (e.g., “Do they fall within the same or into different cycles?”). Importantly, the physical timing between stimuli is identical over trials and behavioral performance is set at a given psychometric threshold, such that the participants’ percept mainly depends on the neural timing of oscillations.

## 4 OSCILLATORY BURST EVENT ANALYSES SUGGEST DISCRETE EVENTS UNDERLIE WORKING MEMORY, THOUGHT, AND MOTOR ACTIONS

We have so far discussed the evidence for discrete activity, where sensory and attention processes use discrete windows of activity as a basic building block. Here, we will review findings from nonhuman primates and humans, in which novel burst analysis have been applied to reveal power dynamics unfolding on single trials (Box 2). This work suggests that cognitive processes, such as associative thought, word comprehension, and prospective functions, such as motor planning and working memory, are supported by similar dynamics that consist of brief bursts (~50–200 ms) of activity (Feingold et al., 2015; Khanna & Carmena, 2017; Kucewicz et al., 2017; Little et al., 2019; Lundqvist et al., ,^2016^, ^2018^, ^2020^; Shin et al., 2017). Bursts in the gamma (> 40 Hz) and beta range (~20 Hz) are related both to the maintenance of working memory information and to motor plans, as well as to the executive control processes of prioritizing or clearing out working memory content (Lundqvist et al., 2018), or canceling memory associations and motor actions (Castiglione et al., 2019; Hannah et al., 2020; Jana et al., 2020).

BOX 2 **Bursting analysis pipeline**

1. Compute time‐frequency power maps for each trial
2. Calculate *Mean* and *Noise* estimate for each trial and frequency
  - *Mean* and *SD* in pre‐trial baseline
  - *Median absolute deviation* across entire trial
3. Threshold single‐trial power maps @ THR (THR =Mean + 2 x *Noise* for 3 cycles per frequency)
4. Sparse “burst” / “no burst” time‐frequency maps for each trial
5. (integration over trials →burst rate in %)
6. Some methods include a final step in which two‐dimensional Gaussians are fit around each burst in the time‐frequency representations. This provides the central frequency of each burst.

### 4.1 Working memory encoding and control

Starting with the encoding and maintenance of visual working memory items, recent data from the prefrontal cortex of monkeys suggest that these functions are supported by brief beta and gamma bursts of activity (Lundqvist et al., 2016, 2018). Several earlier studies had implicated a critical role for gamma oscillations in the encoding of working memory items, where the magnitude of gamma power correlated with the number of items encoded and maintained (Honkanen et al., 2015; Howard et al., 2003; Roux et al., 2012). Single‐trial analysis, inspired by predictions from a computational model (Lundqvist et al., 2011), provided more direct evidence and several new insights into the mechanistic role of gamma oscillations.

**FIGURE 2:**
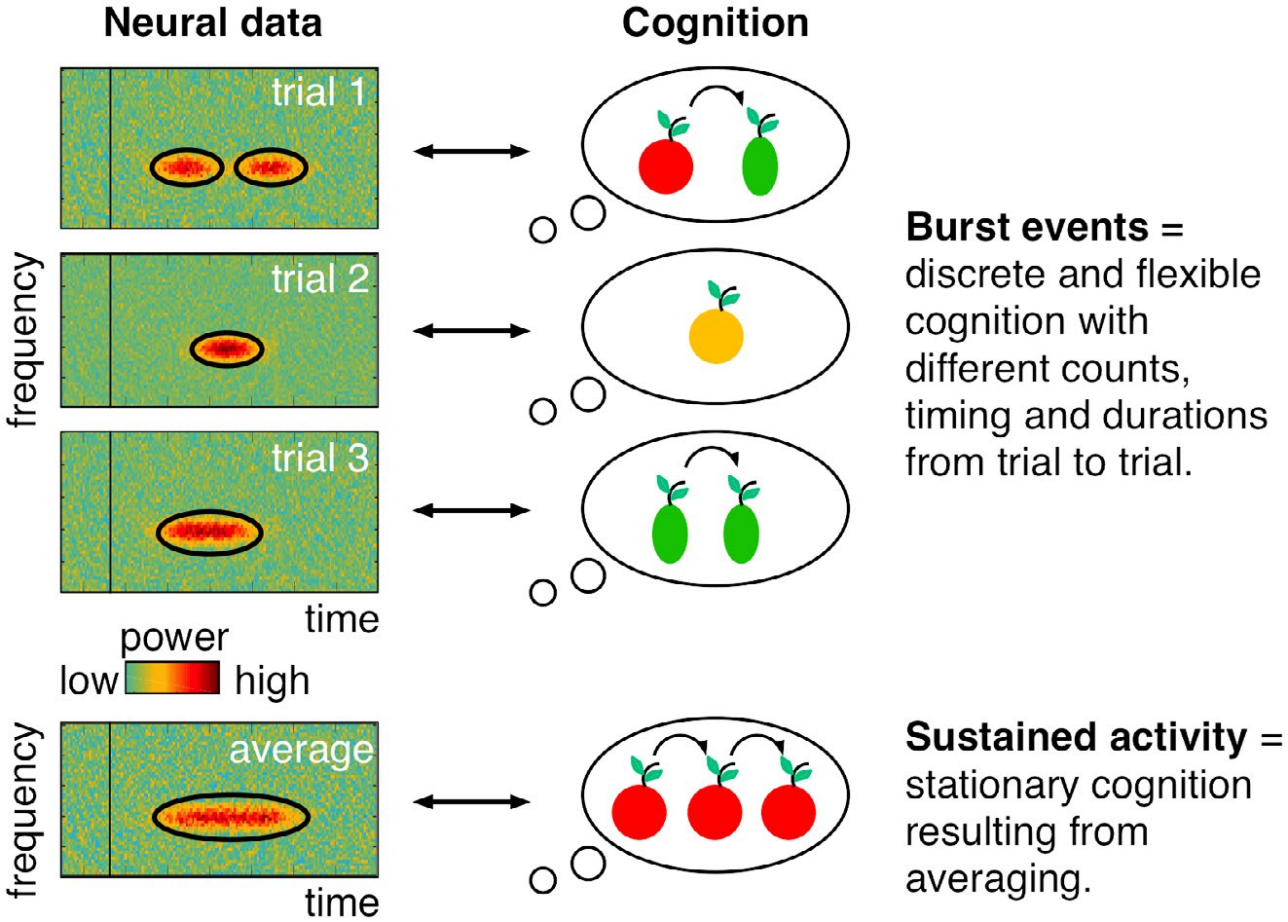
Oscillatory burst events and higher cognition. Schematic depiction for the relationship between burst events and cognitive flexibility. The burst event analysis enables the detection of transient high‐signal epochs (= bursts) that can happen at different rates and times, and with different durations from trial‐to‐trial. It suggests that in many cases, sustained oscillatory activity represents an analysis artifact from averaging over trials. Moreover, burst event dynamics implement flexible computations into models of working memory, thought and action. In such models, one can process more than one item at different time points and for different durations during a memory delay epoch. In contrast, the classic account based on sustained activity does not support such flexible working memories

The tested model was capable of storing multiple items by multiplexing them in time. Each simulated activation and reactivation of an item was accompanied by a gamma burst and high rate spiking selective for that item. In between activations the information related to a specific item was instead imprinted in plastic synapses by the spiking. Since these imprints slowly decayed, maintenance of item‐specific information was dependent on periodic reactivations and thus manifested as intermittent bursts in the gamma frequency range. The interplay between the decay of synaptic plasticity and the reactivations thus sets a limit to the number of items that could be held by the network (Lundqvist et al., 2011). Finally, during time periods in which there was no item reactivation, there were instead bursts of beta activity. The model thus provided several testable predictions that benefited from single‐trial analysis (Figure 2; see also the special section “Advantages of burst analyses”).

First, gamma and beta oscillations were predicted to happen in brief bursts during maintenance, and not in a sustained manner. This was tested and supported by burst extraction analysis of the local field potentials (Lundqvist et al., 2016; see Box 2).

Second, the gamma bursts should co‐occur with the spiking of neurons, which selectively code for the items that were maintained. Importantly, the gamma bursts were not strongly time‐locked to trial events but appeared scattered throughout the trials seemingly at random. Only when the time of their occurrence was averaged across trials did a pattern emerge where gamma burst rates were high at times of encoding and readout and intermediate (but elevated relative pre‐encoding) during maintenance periods. Congruent with the model, beta bursting showed the mirror opposite pattern and was suppressed during encoding and readout, with intermediate (but reduced relative pre‐encoding) levels during maintenance. The fact that bursts were not strongly time‐locked to task events meant that the gamma burst‐spike relationship could be investigated on single trials (see the special section “Advantages of burst analyses”). By analyzing, on each trial when the bursts occurred we were able to select spikes that happened inside or outside of the burst events. This revealed that spiking increased during gamma bursts and to a larger degree reflected the items stored (Lundqvist et al., 2018).

Finally, the single‐trial analysis also provided details about the observed correlation between working memory load and gamma power. There are in principle several ways in which power could change between conditions. There could be baseline shifts in power, increased number of bursts, increased duration of bursts, or increased power during the bursts. Due to increased multiplexing with memory load, the tested model predicted that the number of gamma bursts should increase but not the other possible factors such as their duration. This was supported by the burst analysis (Lundqvist et al., 2016). This result has recently been confirmed with human MEG data, which revealed discrete burst events in the alpha band and from inferior parietal cortex during a multiple‐object tracking task (Wutz et al., 2020). Interestingly, the number of bursts (and not their duration or power) breaks down when processing is above capacity or when objects are summarized into groups (and not as individual items).

Also the executive functions of working memory were supported by beta and gamma burst dynamics. We observed that gamma bursting “ramped up” (and beta ramped down) before the test epoch when the retained information had to be actively used to complete the task. Similar ramp‐ups in spiking or gamma have been observed when the timing of the test onset is predictable (Hussar & Pasternak, 2010). Here, in a two‐item WM task where the items where tested one after the other, the “ramp‐up” of information was specific to the item that was about to be tested, and not a general anticipation signal (Lundqvist et al., 2018). Beta bursting reflected this selective control and predicted behavioral errors. Instead, beta “rebound” following the trial could be linked to clear out of working memory contents to avoid interference across trials. Thus, a single mechanism, the balance between beta and gamma bursting, could potentially explain how information is regulated during encoding, retention, read‐out, and WM reallocation (Bastos et al., 2018; Lundqvist et al., 2016, 2018; Miller et al., 2018).

Interestingly, these patterns of gamma and beta bursting were seen not only in prefrontal cortex but throughout the cortical hierarchy (Lundqvist et al., 2020). However, a key difference between regions was that the central frequency of bursts was gradually higher the higher in the cortical hierarchy the LFPs were recorded from. This could have important implications for how regions of the brain communicate as it sets the duration of the discrete packages sent. The instantaneous frequency of oscillations (Box 1) has been investigated during working memory performance. This revealed that the central frequency of beta oscillations during the working memory delay increased with working memory load. For each added item, the frequency of the beta oscillations in the following delay got elevated (Noguchi & Kakigi, 2020; see also Lundqvist et al., 2018 for a similar pattern in central frequency of bursts).

Similar to the inhibitory role in working memory, bursts in the beta range help to control associative thought processes. This was investigated in a novel experiment where the human participants first learned paired word associations which they should complete when cued with one of the words in a pair. However, in a subset of trials they received a stop cue informing them to not perform the learned association (Castiglione et al., 2019). The stop cue elicited a brief increase in frontal beta, similar to the prefrontal beta involved in the working memory control discussed above. The power of beta was greater on trials in which the halted association was successful. Thus, the control of associative thoughts and working memory may be based on similar neural mechanisms (Schmidt et al., 2019).

It has been suggested that working memory evolved from motor planning, as similar control functions are needed to maintain and execute motor plans (O’Reilly & Frank, 2006). Working memory would, in this view, be based on an analogous prefrontal‐thalamic‐basal ganglia circuitry, but located in anatomically more anterior parts of the brain, controlling more abstract information. In support of this view, we will see below that there are striking similarities in the beta and gamma burst dynamics between working memory, movement planning, and control.

### 4.2 Motor planning and inhibition

Gamma and beta oscillations have long been implicated in motor behavior and are modulated by movements in both basal ganglia and in motor cortex (Feingold et al., 2015; Kilavik et al., 2013; Sanes & Donoghue, 1993). Gamma oscillations are typically seen at the execution of the movement and beta oscillations during periods, in which movements are withheld. Moreover, beta often rebounds just after a movement has been executed. These correlates have led to several interpretations of beta as either involved in movement planning (Little et al., 2019; Rhodes et al., 2018; more), preserving status quo (Engel & Fries, 2010), error processing (Little et al., 2019; Torrecillos et al., 2015), or inhibiting motor responses (Kononowicz & van Rijn, 2015; Pogosyan et al., 2009; Zhang et al., 2008). Single‐trial burst analysis sheds now new light on these interpretations. First of all it suggests that there are high power events in both the beta and gamma frequency ranges occurring in all behavioral modes (Feingold et al., 2015). In contrast to observing trial averages, it is not directly obvious from the spectro‐temporal representations of neural data from single trials when for instance the go cue is given. Instead, the bursts in both frequency ranges are scattered throughout the trials. However, accumulating the time of burst events over trials gives back and explains the behavior observed in trial‐averaged power.

Due to this distribution in time of beta bursts it is possible to look at single‐trial correlates of their function, for example, how the occurrence of beta bursts impact velocity of movements or movement onset. By analyzing in particular the timing of beta bursts it has been possible to gain insights into their role in motor behavior. Several studies indicate that reaction times are dictated by beta bursts, with the presence of beta bursts delaying onset of movement in humans and animals (Khanna & Carmena, 2017; Leventhal et al., 2012; Little et al., 2019). In addition, the amount of beta bursts present during movements themselves seems to slow down their trajectories on a trial‐to‐trial level (Lofredi et al., 2019). These findings support the notion that beta plays a similar inhibitory role in motor behavior as in the working memory studies above. While the notion that beta simply acts as a break in neural systems may seem like an oversimplification, there could be many ongoing processes related to sensory processing, predictions, attention, and so on that needs to be inhibited at different times during an experiment. Presently motor beta bursting or power increases are tied to a number of cognitive processes including saliency evaluation, the processing of relevant cues and errors (Leventhal et al., 2012; Little et al., 2019; Saleh et al., 2010; Schmidt et al., 2019; Torrecillos et al., 2015). Alternatively, beta has been suggested to correspond to the large‐scale communication, rather than inhibition, related to these various processes (Kilavik et al., 2013).

Further evidence for the inhibitory role of beta bursts in cognition and action execution comes from tasks in which a planned action suddenly has to be stopped. In these Go‐NoGo tasks, subjects had to execute a cued motor command with a delay (Swann et al., 2009; Zhang et al., 2008). However, on a small subset of trials the subjects received a stop cue just prior to the planned movement instructing them to halt the action. Following the stop cue there was a transient increase in prefrontal beta, which was greater when subjects successfully manage to abort the planned action. Recent burst analysis revealed there was a cascade of events starting with the onset of a beta burst in prefrontal cortex, continuing down into basal ganglia and then, leading to skeletomotor suppression (Chen et al., 2020; Hannah et al., 2020; Jana et al., 2020). This work also demonstrates the yet untapped potential of burst analysis. As it yields a Boolean series (burst or no burst) for each frequency range and cortical region of interest, with well‐defined burst onset times, it lends the potential to study cascades of activity both across regions and frequencies. We envision that this will be a significant future contribution of burst analysis.

While burst analysis highlights the nature of cognitive processes, it can also be a useful clinical tool as it allows more precise diagnostics of abnormal dynamics in cognitive diseases, such as Parkinson's disease (Follett et al., 2010; Kühn et al., 2006; Lofredi et al., 2019; McCarthy et al., 2011; Schmidt et al., 2019; Tinkhauser et al., 2017; Vinding et al., 2020). As in the above working memory experiments, it is possible to examine in greater detail the underlying mechanisms for the observed difference in beta power between patients and healthy controls (see the special section “Advantages of burst analyses”). Such analysis has revealed that the abnormal beta power observed in patients have different underlying cause depending on brain region. Whereas in sensory motor areas it is the rate, not the duration of beta bursts that are changed, in basal ganglia it is primarily the abnormally long duration of bursts that correlate with disease (Tinkhauser et al., 2017; Vinding et al., 2020). This more precise diagnosis with single‐trial analysis will help early detection. It may also provide more accurate deep‐brain stimulation treatment. Since patients typically adapt to continuous stimulation and since there are other side effects, more precise protocols with the stimulation triggered by the beta bursts one wishes to suppress, could be advantageous (Schmidt et al., 2019; Swann et al., 2018).

## 5 SPECIAL SECTION: “ADVANTAGES OF BURST ANALYSES”

Extracting high power events, or bursts, from time series data provides several advantages.

1. It gives a more accurate representation of the ongoing cortical dynamics and suggests that they are more transient and variable than suggested by traditional power analysis. It, therefore, reveals the fundamental nature of the dynamics under investigation (Lundqvist et al., 2016). This in turn opens up the need for novel analytical tools. When the cortical dynamics are discrete and evolving much faster than the behavioral epochs of the studied task, it cannot be assumed that trial events are aligned. Taking this into account can reveal that the brain switches between a low number of states in a stereotypical order on each trial, but due to jitter in timings across trials it looks like a continuous stream of numerous states when averaging across trials (Vidaurre et al., 2019).
2. It provides a single‐trial representation of the oscillatory dynamics. Thus, the timing of events can be examined on single trials and correlated with behavior or other neurophysiological measures. This includes reaction times (Jana et al., 2020; Khanna & Carmena, 2017), error trial analysis (Jana et al., 2020; Little et al., 2019; Lundqvist et al., 2018), and spike‐field relationships (Lundqvist et al., 2018).
3. It gives the duration number and power of such events. There may be several underlying factors why power differs between two conditions. Power may change due to increased rates of bursts, due to longer burst durations, or due to different power levels during or outside the bursts. Thus it reveals the mechanisms by which power changes and provides important constraints to models. Examples of this include comparing different working memory loads (Lundqvist et al., 2016) or patients with Parkinson's disease to healthy controls (Tinkhauser et al., 2017; Vinding et al., 2020).
4. The frequency within the band of interest changes across conditions or subjects. Some burst extracting methods (see Kucewicz et al., 2014 and Lundqvist et al., 2016 for two alternative methods) also estimate the central frequency of each burst using the time–frequency representations. Thus, they can be used to assess the instantaneous frequency.

## 6 CONCLUSIONS/OPEN QUESTIONS

We have reviewed evidence that new analytical methods, which are more sensitive to the real‐time dynamics of neurophysiological measures, demand new theories of cognition. Different methods for examining single‐trial dynamics of neural activity provide substantial evidence that the brain operates using discrete windows for sensory processing, integration, and communication. Evidence for these discrete events can be observed throughout the cortical hierarchy, including sensory, prefrontal, and motor output areas, as well as in subcortical regions. They act as basic building blocks in a wide range of cognitive processes including sensory individuation and integration, top‐down attention, as well as prospective functions such as working memory, motor planning, and associative thought. What is the underlying function of such discrete dynamics? We argue it aids the brains dual requirements for flexibility and stability (Sandberg, 1963), both from a cognitive and a dynamical systems perspective.

In terms of cognitive processes, this tradeoff between flexibility and stability can be described by our dual needs of reaching stable cognitive outputs in noisy environments but at the same time stay vigilant to small but important changes in them. Packaged processing, in which each discrete window sets a time limit on each cognitive action (i.e., sensory integration, motor output, and re‐sampling) helps to achieve both these goals virtually at the same time. As an example, devoting a long enough time window for sensory integration of a certain stimulus will help to reach stable identification. Moreover, the punctuation of the integration process by the end of the window will give the brain a reset, that is, a chance to switch to a new cognitive thread, and prevent inflexible and obsessive behavior. By devoting a fixed time window to each process, cognition may concurrently resolve conflicts between stimulus read‐out versus perceptual synthesis, attentional exploitation versus exploration (see also e.g., Fiebelkorn & Kastner, 2019), and memory maintenance versus its volitional control (e.g., via temporal multiplexing; Panzeri et al., 2010).

Discrete processing windows may seem similar to having a sliding window of sensory integration, but this is only the case if the brain is viewed as a passive receiver (VanRullen & Koch, 2003). However, multiple sources of evidence suggest that the brain engages in “active sensing,” based on sensory evidence plans and conducting motor commands through goal‐driven behavior, such as decreasing uncertainty (Schroeder et al., 2010; Yang et al., 2016). In this view, discrete time windows, supported by neural oscillations, add flexibility, since they can be phase reset by explorative motor commands, for example, to time sensory integration to the onset of a saccade (Bartlett et al., 2011; Ito et al., 2011; Wutz, et al., 2018). Indeed, the phase and frequency analyses, discussed here, suggest that phase resets in the alpha band are tied to the successful individuation of objects (Wutz et al., 2014) and that actively adjusting the central frequency of alpha oscillations aids individuation versus integration (Wutz, Melcher, et al., 2018). Likewise, as neural oscillations reflect periodic shifts between excited and inhibited states, they may provide distinct windows of sensory integration and evaluation (continue to sample current location vs. saccade to new location) also in visual attentional processes (Fiebelkorn & Kastner, 2019). The inherent drawback to this approach is that it sets an upper limit on the cognitive bandwidth. However, the reliability of processing, that is, not confusing information belonging to distinct cognitive threads, may be facilitated by operating with discrete packages of activity. Accordingly, discrete packages have also been suggested to improve stability of cortical long‐range communication and allow the brain to utilize spike‐time coding, because the time of the packet onset can be used as a reference (Herman et al., 2013; Luczak et al., 2015).

Discrete windows of activity may also contribute to dynamic stability and help to control runaway excitation. The brain needs to handle inputs that vary in amplitude by several orders of magnitude. It also needs to be stable to handle vast changes in the inner excitability due to the dynamic activation of connected brain regions, general arousal, modulation of neurotransmitters etc. Sampling the environment or cognitive threads intermittently and punctuating the resulting ignited recurrent excitation between samples prevent runaway excitation by setting an upper limit on the amount of spiking activity that can be recruited before activity gets suppressed again. Neural oscillations generated by feedback inhibition have been demonstrated to increase stability: as long as the inhibition is strong enough to eventually overcome the excitation it allows for a wide range of excitatory activity (Lundqvist et al., 2010). Packaging oscillations into bursts serves a similar function. As we discussed, it may also set a bandwidth limit on cognition and behavior, but that may be two sides of the same coin: processing capacity has been sacrificed for stability.

The evidence we have provided here comes from studying oscillatory neural activity in humans (often with MEG recordings) and nonhuman primates. The recent ability to record and analyze thousands of neurons simultaneously in rodents has accumulated analogous evidence for discrete dynamics as foundations to cognitive and sensory processing (Engel et al., 2016; Luczak et al., 2009, 2015; Stringer et al., 2019). This is seen as transient periods of coordinated high rate activity of entire neural ensembles followed by periods of relative silence. This activity is often revealed by novel analytical methods including clustering, methods for detecting hidden population states, and dimensionality reduction methods (Abeles et al., 1995; Stringer et al., 2019; Vidaurre et al., 2019). Since neural oscillation recordings reflect the coordinated activity of millions of single neurons, they in fact reflect the low dimensional activity of the brain, too. Since both spike and field analysis seem to suggest similar dynamics, we believe the two—up until now largely separated fields—will merge closer together with the increased ability to record a large number of neurons simultaneously. This view is supported by studies that analyze concomitant neural oscillations and spiking, suggesting that spikes and oscillatory burst events follow each other on single trials (Lundqvist et al., 2016, 2018). Thus it appears likely that the view laid forward here, that the brain builds its functions on discrete dynamics, will continue to accumulate evidence. The next step is experiments directly shaped by, and directly testing, this emerging view. We also envision that the analysis tools, discussed here, will be used to describe how discrete events propagate across the cortical hierarchy to form chains of sensory percepts or motor commands. The coming decade of neuroscience will likely detail the mechanisms of how these discrete dynamics are used to form flexible cognition.

## AUTHOR CONTRIBUTIONS

**Mikael Lundqvist:** Conceptualization; Investigation; Methodology; Writing‐original draft; Writing‐review & editing. **Andreas Wutz:** Conceptualization; Investigation; Methodology; Visualization; Writing‐original draft; Writing‐review & editing.
